# An Examination of the Latent Constructs in Risk Tools for Individuals Who Sexually Offend: Applying Multidimensional Item Response Theory to the Static-2002R

**DOI:** 10.1177/10731911221076373

**Published:** 2022-02-18

**Authors:** Sébastien Brouillette-Alarie, Seung C. Lee, Nicholas Longpré, Kelly M. Babchishin

**Affiliations:** 1 Université de Montréal, Quebec, Canada; 2 Public Safety Canada, Ottawa, Ontario, Canada; 3 Edge Hill University, Ormskirk, UK; 4 Carleton University, Ottawa, Ontario, Canada

**Keywords:** item response theory (IRT), multidimensional, Static-2002R, risk assessment, sexual offending, factor analysis

## Abstract

Multidimensional item response theory (MIRT) was used to study the construct validity of the Static-2002R, an actuarial scale for the assessment of reoffending among adult men who sexually offended. Using a sample of 2,569 individuals with a history of sexual crime, exploratory factor analysis (EFA) extracted three factors: Persistence/Paraphilia, General Criminality, and Youthful Stranger Aggression. MIRT confirmed the factor structure identified in the EFA model and provided item-level data on discrimination and difficulty. All Static-2002R items showed moderate to very high discrimination and covered a wide range of risk levels (i.e., difficulty). MIRT analyses attested to the construct validity of the scale, as no items were identified as problematic and the resulting factor structure was consistent with that of earlier studies. Considering the stability of results pertaining to the factor structure of the Static-2002R and the advantages of dimensional scoring, we recommend the integration of dimensional scores in the scale.

Actuarial scales for the assessment of risk for criminal reoffending have typically prioritized predictive accuracy over construct validity. Following the documentation of the pitfalls of unstructured clinical judgment in the 1950s and after (Dawes et al., 1989; Grove et al., 2000; Hanson & Morton-Bourgon, 2009; Meehl, 1954), the fields of correctional psychology and criminology turned to actuarial assessment to reliably measure the level of risk of offending populations (Harris & Hanson, 2010). Actuarial scales rely on a mechanical combination of predictors and are considered “atheoretical” because they often contain a list of risk-relevant items not organized by dimensions or, alternatively, comprise conceptual dimensions that were not empirically validated by psychometric analyses (Andrews & Bonta, 2010; Bonta, 1996; Brouillette-Alarie & Lussier, 2018).

Among risk tools developed for individuals adjudicated for a sexual offense, the Static-99R (Hanson & Thornton, 2000) and Static-2002R (Hanson & Thornton, 2003; Helmus et al., 2012) are the most used internationally and inform a number of legal decisions made with this population (Archer et al., 2006; Bourgon et al., 2018; Jackson & Hess, 2007; Kelley et al., 2020; Neal & Grisso, 2014). The Static-99R comprises 10 commonly available items related to demographic characteristics, criminal history, and victim choice. The Static-2002R improves coding consistency and conceptual clarity compared with the Static-99R by organizing its 14 items into five subscales: Age at Release, Persistence of Sexual Offending, Sexual Deviance, Relationship to Victims, and General Criminality. The General Criminality subscale and age item of the Static-2002R can be combined to score the *Brief Assessment of Recidivism Risk–2002R* (BARR-2002R), a scale that predicts violent and general recidivism better than the Static-99R and Static-2002R and screens for antisocial tendencies among adult men who sexually offended (Babchishin et al., 2016). Both the Static-99R and Static-2002R, as their name implies, are static risk tools, which means that they only comprise static risk factors. Static risk factors are considered easier to score than dynamic factors because the data on which they rely are easily accessible in correctional files and are seen as devoid of subjectivity (Bonta, 2002; Gendreau et al., 1996). They have, however, a limited ability to inform treatment, determine parole conditions, and assess positive or negative changes in offenders’ lives (Andrews & Bonta, 2010; Bonta, 1996, 2002; Douglas & Skeem, 2005; Gendreau et al., 1996).

In the last 20 years, a number of studies have sought to identify latent constructs in static risk scales for individuals with a history of sexual crime by using latent variable models, such as factor analysis (e.g., Roberts et al., 2002; for a review, see Brouillette-Alarie et al., 2016). Clarifying the construct validity of static risk tools has many potential advantages for the field. First, it offers insight into why certain scales predict certain outcomes better than others, as this is dependent on the constructs they assess and how each construct is weighted in these scales. This, in turn, can help evaluators integrate the potentially conflicting results of similar risk scales when multiple tools are available for the same population (Barbaree et al., 2006). Second, understanding the constructs implicit in risk tools can improve predictive accuracy. Specifically, when the constructs are known, it is possible to improve the reliability and validity of their assessment using standard psychometric methods and, therefore, improve the predictive accuracy of the scale (Brouillette-Alarie et al., 2016). Finally, construct-level approaches maximize the clinical relevance of existing scales by focusing on psychological dimensions, facilitating the identification of the “source” of the risk. This enables static risk tools to give insight into the assessment of potentially changeable constructs; although the items found in these scales are not modifiable through intervention, the latent psychological constructs they represent are amenable to change (Babchishin & Hanson, 2020; Beech & Ward, 2004; Brouillette-Alarie et al., 2018). Evaluations that address psychological features are generally better received by clinicians, practitioners, and decision makers than those that only delineate the level of risk (Mann et al., 2010).

Multiple studies investigated the latent constructs of the Static-2002(R). Boughner (2010), Ennis et al. (2011), and Langton et al. (2007) studied the factor structure of the original Static-2002; Jung et al. (2017) studied the Static-2002R; and Brouillette-Alarie et al. (2016) and Rohrer (2019) studied nonredundant items from both the Static-99R and Static-2002R. These studies identified at least two constructs consistent with theoretical models of sexual recidivism (e.g., Doren, 2004): sexual criminality and general criminality. Sexual criminality was typically defined by items related to the number of sexual offenses and indicators of paraphilic sexuality (e.g., child victims, noncontact sexual offenses), while general criminality was defined by items that reflect the magnitude, violence, and diversity of criminal careers (e.g., number of prior sentencing occasions, prior nonsexual violence). In addition to these two constructs, others were found in Static-99R and Static-2002R items. Although there is no clear consensus on the number and nature of additional constructs, they seem to be mostly related to age, relationship history, violence in the index offense, and the sexual abuse of unrelated and stranger victims.

Convergent validity analyses have also been carried out to clarify the psychological meaning of these constructs (Brouillette-Alarie et al., 2018; Brouillette-Alarie & Hanson, 2015). The sexual criminality construct was correlated with atypical sexual interests/paraphilias (especially pedophilia), emotional identification with children, and grooming offending strategies. The General Criminality construct was associated with numerous features of antisocial personality disorder (American Psychiatric Association, 2013) and psychopathy (Hare, 2003). The third construct, “Youthful Stranger Aggression,” was correlated with offense seriousness and sexual sadism and was therefore interpreted as a motivation to harm victims. Sexual criminality was more prevalent in individuals having sexually abused children, while the other two, General Criminality and Youthful Stranger Aggression, were more common in individuals who have sexually assaulted women. All three constructs predicted sexual recidivism, but only General Criminality and Youthful Stranger Aggression predicted nonsexual types of recidivism (Brouillette-Alarie et al., 2016, 2018).

## Item Response Theory (IRT) as a Tool to Study Construct Validity

Although the aforementioned studies offered valuable insight into the latent constructs of assessment tools used to predict reoffending among men with sexual offenses, they were anchored in classical test theory (CTT) models, that is, exploratory factor analysis and confirmatory factor analysis (EFA/CFA). Scholars from the field of education have long advocated for the use of IRT models, as they are less sensitive than CTT models to circular dependency, that is, dependence on the overall performance of the validation sample (de Ayala, 2009). IRT was introduced in the 1950s and 1960s by Lord (1953) and Rasch (1960) to better assess item discrimination and difficulty, and create sample-free measures (Osteen, 2010). IRT models assume that an examined latent trait, denoted theta (θ), is unidimensional and analogous to the “true score” in CTT (de Ayala, 2009). Thus, a response to an item is influenced by both the qualities of the participant and the properties of the item. The graph of the relation between the ability score of a person and the probability that this person will endorse an item is called the item characteristic curve. IRT models, unlike CTT, do not assume a linear relationship among these, so that the item characteristic curve takes the form of an S-shaped curve (Reid et al., 2007). This feature is relevant for criminological actuarial scales because most of their items are dichotomous and, thus, better fitted by an S-shaped curve than a linear relationship (akin to how logistic regression better fits dichotomous outcomes than linear regression).

Examining the structure of a scale via IRT requires that the scrutinized construct be unidimensional (de Ayala, 2009). That is, IRT assumes that item covariations arise from a single underlying dimension, and its violation leads to unstable IRT model parameter estimates and weak model fit (de Ayala, 2009; Reise et al., 2005, 2011, 2015). This is problematic because risk scales for individuals with a history of sexual crime are multidimensional. To circumvent the unidimensionality assumption, multidimensional item response theory (MIRT) was introduced in the 1970s to analyze scales that are multidimensional (Reckase, 1972). MIRT is a probabilistic model designed to measure an individual’s likelihood of responding to a specific item based on item parameters and multiple latent traits (Reckase, 2009). This technique allows us to model multiple latent traits simultaneously without raising measurement error and model instability (Reckase, 2009).

Compared with factor analysis, MIRT has numerous advantages: It overcomes the item–person confound of CTT models, allows more accurate treatment of the standard error of measurement, enables the use of dichotomous and ordinal data, and provides indices on how well each item performs (Osteen, 2010). MIRT models are tools that can be used in conjunction with factor analysis models to better triangulate evidence via the comparison of results of nonredundant analytical procedures. Note that unlike traditional EFA and CFA, item factor analysis (see Wirth & Edwards, 2007) should not be considered as CTT and shares many advantages inherent to the use of MIRT models.

To our knowledge, only one study used MIRT to explore the latent constructs of risk scales for individuals with a history of sexual crime. Allen and Pflugradt (2014) explored the factor structure of the Static-99 using Normal Ogive Harmonic Analysis Robust Method (Fraser & McDonald, 1988), a nonlinear, MIRT-based factor analysis model. They found three constructs similar to those found by Brouillette-Alarie et al. (2016). Allen and Pflugradt’s (2014) sample size (*N* = 451), however, was low. Sample sizes of 1,000 (Jiang et al., 2016) or 2,000 (Forero et al., 2009) are recommended for MIRT, as small sample sizes can lead to unstable model fits and unreliable item parameters. In addition, their analysis was conducted on the Static-99 rather than the Static-2002R, which is the most up-to-date version of the scale.

## Current Study

We applied an MIRT model to investigate the latent structure of the Static-2002R, a risk tool for adult men who sexually offended, and compared results to what is found in traditional factor analytic models. Because sample peculiarities (incarceration vs. community, high vs. low risk, etc.) are of foremost importance in risk tool validation and use, we employed a sample of 2,569 participants that maximized the chances of being representative of multiple correctional settings while satisfying the sample size requirements of MIRT. In turn, MIRT models maximized the chances that our results were sample-agnostic and, thus, generalizable to diverse correctional settings. In the following section, we report how we determined our sample size, all data exclusions, all manipulations, and all measures in the study.

## Method

### Sample

This study used datasets from a project involving the re-norming of the Static-99 and Static-2002 (for additional information on the samples and data preparation, see Helmus, 2009). These datasets constitute a nonexhaustive collection of contemporary validation studies of the Static-99, with some having Static-2002 data. These samples should be broadly representative of typical settings in which the Static-99R and Static-2002R are used. To be included in the current study, samples from the re-norming project were required to have information on all Static-2002R items. In total, seven samples were available (*N* = 2,569).

Descriptive information of the included datasets can be found in Table 1. Four samples were from Canada; the remaining samples were from Denmark (*k* = 1), the United Kingdom (*k* = 1), and the United States (*k* = 1). Three samples consisted of routine correctional samples (i.e., relatively unselected, expected to be fairly representative of the population of individuals convicted for a sexual offense), either from prisons (Bigras, 2007; Boer, 2003) or community supervision (Hanson et al., 2007). One sample was a treatment sample (Harkins & Beech, 2007), whereas the rest were more specialized, including a sample evaluated for civil commitment (Knight & Thornton, 2007), receiving specialized forensic psychiatric evaluations (Bengtson, 2008), or detained in federal prison until the expiration of their sentence (Haag, 2005). In the full sample, the average Static-99R score was 3.1 (*SD* = 2.6) and the average Static-2002R score was 4.3 (*SD* = 2.7). For reference, a score of 3 on the Static-99R corresponds to the 66th percentile, while a score of 4 on the Static-2002R corresponds to the 64th percentile (Hanson et al., 2012). The average age at release was 39 years old (*SD* = 12).

**Table 1:** Descriptive Statistics of Samples.

Variables/studies	Bengtson (2008)	Bigras (2007)	Boer (2003)	Haag (2005)	Hanson et al. (2007)	Harkins & Beech (2007)	Knight & Thornton (2007)	Total sample
*n*	308	425	296	190	702	182	466	2,569
Type of sample	Forensic psychiatric evaluations	Routine CSC	Routine CSC	Detained until end of sentence	Routine community supervision	Prison and community treatment	Civil commitment	NA
Country	Denmark	Canada	Canada	Canada	Canada	The United Kingdom	The United States	NA
Release period	1978–1995	1995–2004	1976–1994	1995	2001–2005	1994–1998	1957–1986	1957–2005
Median year of release	1986	1999	1990	1995	2002	1995	1970	1995
Recidivism criteria	Charges	Charges	Convictions	Convictions	Charges	Convictions	Charges	NA
Follow-up in years, *M* (*SD*)	16.2 (4.3)	4.6 (1.9)	13.3 (2.1)	7.0 (0.0)	3.5 (1.0)	10.4 (1.1)	8.6 (2.6)	8.0 (4.9)
Sexual recidivism rate	34.1%	5.9%	8.8%	24.7%	8.1%	13.7%	28.1%	16.2%
Nonsexual violent recidivism rate	23.4%	9.9%	17.6%	NA	8.8%	10.4%	24.9%	15.3%
Any recidivism rate	64.6%	23.3%	51.7%	NA	27.9%	35.2%	54.9%	40.2%
Age at release in years, *M* (*SD*)	32.5 (10.4)	42.7 (11.9)	41.2 (12.5)	36.7 (9.7)	41.6 (13.2)	43.5 (12.7)	36.1 (11.4)	39.4 (12.5)
Static-99R, *M* (*SD*)	3.8 (2.4)	2.1 (2.3)	2.8 (2.8)	4.1 (2.2)	2.4 (2.4)	2.1 (2.6)	4.6 (2.4)	3.1 (2.6)
Static-2002R, *M* (*SD*)	4.6 (2.4)	3.4 (2.5)	3.9 (2.7)	5.7 (2.3)	3.5 (2.5)	3.6 (2.8)	6.1 (2.5)	4.3 (2.7)

*Note.* CSC = Correctional Service of Canada.

### Measures

#### Static-2002R

Similar to the Static-99R, the Static-2002R (Hanson & Thornton, 2003; Helmus et al., 2012) is an empirical actuarial risk assessment tool for adult males who sexually offended (see also https://saarna.org/). It has 14 items grouped into five main subscales: Age at Release, Persistence of Sexual Offending, Sexual Deviance, Relationship to Victims, and General Criminality. The total score (ranging from −2 to 13) can be used to place offenders in one of five risk categories: Level I—very low risk (scores of −2 to −1), Level II—below average risk (scores of 0 to 1), Level III—average risk (scores of 2 to 4), Level IVa—above average risk (scores of 5 to 6), and Level IVb—well above average risk (scores of 7 or higher) (Hanson et al., 2017). Static-2002R items are identical to Static-2002 items, with the exception of updated age weights.

The Static-2002 was originally developed to improve coding consistency, conceptual clarity, and predictive accuracy compared with the Static-99. Although the Static-2002 was more accurate than the Static-99 (Hanson et al., 2010), revising Static-99 age weights increased its predictive accuracy such that there was no longer a meaningful difference between the Static-99R and the Static-2002R (Babchishin et al., 2012). Both scales, however, contribute incrementally to the prediction of sexual recidivism (Babchishin et al., 2012; Lehmann et al., 2013).

#### Item Preparation

Initial item preparation was required before inputting items from the Static-2002R in statistical analyses. First, we combined items that were part of the same continuum to avoid potential collinearity issues. Specifically, the “any unrelated victim” and “any stranger victim” items were combined into one item on a 3-point scale: 0 = *no unrelated or stranger victim*, 1 = *at least one unrelated victim and no strangers*, and 2 = *at least one stranger victim*. In addition, the “any prior involvement with the criminal justice system” and “prior sentencing occasions for anything” items were combined into one item on a 4-point scale: 0 = *no prior involvement with the criminal justice system*; 1 = *at least one prior charge, but less than three prior sentencing occasions*; 2 = *three to 13 prior sentencing occasions*; and 3 = *14+ prior sentencing occasions*. Next, we examined potential collinearity (*r* > .80) between items with tetrachoric/polychoric correlations (appropriate for dichotomous and ordinal items; Flora et al., 2012) and found a high correlation between the “high rate of sexual offending” and “prior sentencing occasions for sexual offenses” items (*r* = .95). Thus, the “high rate of sexual offending” item was dropped in this study. In sum, 11 items were entered in the factor analysis and MIRT model (see Appendix A for the full list of items).

### Analytical Strategy

#### Factor Analysis and Dimensionality

To confirm whether the Static-2002R was unidimensional or multidimensional in our sample, an EFA of Static-2002R items was performed using the R statistical program and “*mirt*” package (Chalmers, 2012). EFA guidelines suggested by Brouillette-Alarie et al. (2016) were followed after adapting them to the *mirt* package, which meant (a) using quasi-polychoric correlation matrices; (b) extracting factors using the Metropolis–Hastings Robbins–Monro algorithm (Cai, 2010); (c) rotating factors using an oblique rotation (geomin); (d) assessing factor structure fit with the root mean square error of approximation (RMSEA, should be <.06), comparative fit index (CFI, should be >.95), and Tucker–Lewis index (TLI, should be >.95) (Hu & Bentler, 1999); and (e) determining factor inclusion with factor loadings of at least .40 (Stevens, 1992). In addition to the three fit indices outlined above, the Akaike’s (AIC; Akaike, 1973) and Bayesian (BIC; Schwarz, 1978) information criteria were used. For both criteria, the lower value indicates the better-fitting model. Quasi-polychoric correlations between Static-2002R items were computed using the “*psych*” package (Revelle, 2021).

To verify if a single “risk” construct encompassed the three Static-2002R dimensions, a bifactor model (Reise, 2012) was run in the R *mirt* package. The AIC and BIC of regular factor solutions were compared with those of the bifactor model to see which structure better represented the data. Only these two fit indices were used because when comparing a traditional model with a bifactor model, the CFI and RMSEA will always favor the bifactor model as it is less parsimonious. In contrast, the AIC and BIC consider the number of parameters in the model (i.e., provides a penalty for each additional parameter), allowing to examine the improvement in fit for models with varying parameters, such as the models examined in the current study.

#### MIRT

MIRT was applied to Static-2002R items following the factor structure identified in the EFA. Two-parameter MIRT was performed, which allowed for items to vary not only in their difficulty on the latent trait but also in their capacity to discriminate between persons located at different points on the continuum (Reid et al., 2007). Our sample size (*N* = 2,569) was considered sufficient to provide accurate parameter estimates (Forero et al., 2009; Jiang et al., 2016). Reckase’s (2009) equations (i.e., two-parameter logistics model) were followed to estimate the difficulty and discrimination parameters in MIRT. The logistic model was used:

(1) $$P\left(\chi_{ij}=\;C_{j}|\theta_{i},\alpha_{j},d_{i}\right)=\frac{\sum_{k=1}^{m}\alpha_{jk}\theta_{ik}+\;d_{j}}{1+e^{\left(\sum_{k=1}^{m}\alpha_{jk}\theta_{ik}+\;d_{j}\right)}}.$$

Where i=1,…,N represent the distinct participants; j=1,…,n , the test items; k=1,…,m , step of the graded response item, and suppose that there are *m* latent factors $\theta_{i}=(\theta_{i1},\ldots ,\theta_{im})$ with associated item slopes $\alpha_{j}=(\alpha_{1},\ldots ,\alpha_{m})$ . There are *C_j_* unique categories for item *j*, with intercepts $d_{j}=(d_{1},\ldots ,d_{\left(C_{j}-1\right)})$ .

As seen in Equation 1, slope parameters (α; discrimination) are related to the slope of the surface, which indicates the rate that the probability of a correct response changes from point to point in the θ space. The *d* parameter (intercept) is not a difficulty parameter as seen in unidimensional IRT; it instead indicates the probability of a correct response when all the latent traits are at zero. The negative of the intercept value (–*d*) divided by the multidimensional discrimination parameter (*A*) gives the relative item difficulty related to the corresponding coordinate dimension (see Equation 3).

The multidimensional discrimination parameter, denoted alpha (*A*; see Equation 2), is the degree to which the item has the power to discriminate between individuals who have or do not have the corresponding θ level of latent traits (Reid et al., 2007). According to Baker (2001), discrimination values 0.01 to 0.34 are very low, 0.35 to 0.64 are low, 0.65 to 1.34 are moderate, 1.35 to 1.69 are high, and greater than 1.7 are very high.

(2) $$A_{j}=\sqrt{\overset{m}{\underset{k=1}{\Sigma }}\alpha_{jk}^{2}}.$$

The multidimensional difficulty parameter, denoted beta (*B*; see Equation 3), is the location of the inflection point on the item characteristic curve and usually varies from −3 to 3. The value of *B* has the same interpretation as the *b* parameter for the unidimensional IRT model. Specifically, items located above 0 are considered difficult, whereas values below 0 indicate easier items (de Ayala, 2009):

(3) $$B_{jl}=\frac{-d_{jl}}{\sqrt{\sum_{k=1}^{m}\alpha_{jk}^{2}}}=-d_{j}/A_{j},$$

where *B_j_*_l_ is the step difficulty and *d_j_*_l_ is the step intercept for the *l*th step of the graded response item.

## Results

### EFA

Inter-item correlations ranged from −.27 to .80 (*Mdn* absolute *r* = .25; see Appendix B). The three-dimensional model was the best-fitting one, as its AIC and BIC values were the lowest among all models (see Table 2). In addition, the goodness-of-fit of the three-dimensional model was good, with an RMSEA of .046, a CFI of .987, and a TLI of .965. All the items loaded on at least one factor (factor loadings >.40) and only one item (juvenile and adult sexual offenses) loaded on multiple factors. Furthermore, the ratio between the eigenvalues of the first and second factors was not sufficiently high to conclude that the scale was “unidimensional enough” (Bertrand & Blais, 2004).

**Table 2.** Summary of Factor Structure Fit and Three-Dimensional Factor Loadings (N = 2,569).

Fit indices	One dimension	Two dimensions	Three dimensions	Four dimensions
AIC	36,598.70	35,524.97	34,910.27	34,987.01
BIC	36,762.53	35,747.32	35,185.28	35,308.83
RMSEA	.141	.090	.046	—
CFI	.748	.925	.987	—
TLI	.675	.868	.965	—
	Static-2002R items	Factor 1	Factor 2	Factor 3
Item no.		Persistence/Paraphilia	Youthful Stranger Aggression	General Criminality
1	Age at release	−.231	**.920**	−.053
2	Prior sexual offenses	**.728**	−.009	.399
3	Juvenile and adult sexual offenses	**.535**	**.451**	.197
4	Noncontact sexual offenses	**.565**	−.003	−.044
5	Male victim	**.604**	−.099	−.194
6	Young, unrelated victims	**.783**	.015	−.225
7	Unrelated and stranger victim	.357	**.443**	.044
8	Prior arrest/sentencing occasions	.032	−.054	**.952**
9	Community supervision violation	−.035	.016	**.836**
10	Years free prior to index offense	.119	.336	**.690**
11	Prior nonsexual violence	−.235	− .025	**.859**

*Note.* AIC = Akaike information criterion; BIC = Bayesian information criterion; RMSEA = root mean square error of approximation; CFI = comparative fit index; TLI = Tucker–Lewis index. Factor loadings equal or superior to .40 are in bold.

Similar to Brouillette-Alarie et al. (2016), three factors were found. Factor 1, Persistence/Paraphilia, comprised, in decreasing order of loading: (a) young, unrelated victims; (b) prior sexual offenses; (c) male victim; (d) noncontact sexual offenses; and (e) juvenile and adult sexual offenses. Factor 2, Youthful Stranger Aggression, comprised (a) age at release, (b) juvenile and adult sexual offenses, and (c) unrelated and stranger victim. Finally, Factor 3, General Criminality, comprised (a) prior arrest/sentencing occasions, (b) prior nonsexual violence, (c) community supervision violation, and (d) years free prior to index offense. Persistence/Paraphilia had a correlation of .09 with Youthful Stranger Aggression and of .29 with General Criminality. Youthful Stranger Aggression had a correlation of .25 with General Criminality.

The bifactor model indicated that static recidivism risk in individuals with a history of sexual crime was constituted of three distinct subfactors without sharing a general factor. The fit indices of the three-dimensional bifactor model (AIC = 35,359.95, BIC = 35,588.15) were worse than those of the regular three-factor model (AIC = 34,910.27, BIC = 35,185.28). In addition, the discrimination values of the bifactor model were substantially different from those of a unidimensional model comprising the same items, indicating that multidimensionality needed to be taken into account. Therefore, the Static-2002R appeared to be fully multidimensional in our sample and was not suitable for regular IRT.

### MIRT

Two-parameter MIRT was applied to Static-2002R items following the factor structure identified in the EFA. First, MIRT slopes (α; discrimination) indicated that all Static-2002R items, except for juvenile and adult sexual offenses, performed more effectively (i.e., changed more quickly in probability) in the factor in which EFA classified them (see Table 3). For juvenile and adult sexual offenses, EFA did indicate cross-loading on Persistence/Paraphilia and Youthful Stranger Aggression. Therefore, it was not entirely surprising to see the juvenile and adult sexual offenses item have an important slope in Youthful Stranger Aggression.

**Table 3:** Slopes (α; Discrimination) From MIRT.

	Static-2002R items	Factor 1	Factor 2	Factor 3
Item no.		Persistence/Paraphilia	Youthful Stranger Aggression	General Criminality
1	Age at release	−3.241	**2.279**	0.605
2	Prior sexual offenses	**2.648**	2.598	1.718
3	Juvenile and adult sexual offenses	**0.415**	**2.260**	0.859
4	Noncontact sexual offenses	**0.816**	0.782	−0.078
5	Male victim	**0.966**	0.641	−0.425
6	Young, unrelated victims	**1.320**	1.314	−0.531
7	Unrelated and stranger victim	−0.024	**1.276**	0.296
8	Prior arrest/sentencing occasions	1.228	1.001	**4.904**
9	Community supervision violation	0.083	1.006	**3.026**
10	Years free prior to index offense	<0.001	1.734	**2.629**
11	Prior nonsexual violence	<0.001	<0.001	**2.421**

*Note.* α values of items that had factor loadings equal or superior to .40 in Table 2 are in bold. MIRT = multidimensional item response theory.

Second, as seen in Table 4, all Static-2002R items showed multidimensional discrimination values (*A*; the degree to which the item has the power to discriminate between individuals who have or do not have the corresponding θ level of latent traits) in the moderate to very high range (1.13–5.15). Only noncontact sexual offenses, male victim, and unrelated and stranger victim showed moderate rather than very high discrimination. All General Criminality items were, therefore, very highly discriminant.

**Table 4:** Multidimensional Discrimination (A) and Multidimensional Difficulty (B) Parameters From MIRT.

	Static-2002R items	Multidimensional discrimination	Multidimensional difficulty		
Item no.		*A*	*B*_1_	*B*_2_	*B*_3_
1	Age at release	4.00	−1.58	−0.20	0.26
2	Prior sexual offenses	4.09	0.49	1.28	—
3	Juvenile and adult sexual offenses	2.45	2.01	—	—
4	Noncontact sexual offenses	1.13	2.04	—	—
5	Male victim	1.23	1.43	—	—
6	Young, unrelated victims	1.94	1.32	—	—
7	Unrelated and stranger victim	1.31	−0.92	0.68	—
8	Prior arrest/sentencing occasions	5.15	−0.65	0.26	1.75
9	Community supervision violation	3.19	0.22	—	—
10	Years free prior to index offense	3.15	0.32	—	—
11	Prior nonsexual violence	2.42	0.61	—	—

*Note.* MIRT = multidimensional item response theory.

Third, results in Table 4 showed that all Static-2002R items except age at release, unrelated and stranger victim, and prior arrest/sentencing occasions were difficult (multidimensional difficulty [*B*] > 0), indicating they were reflective of relatively uncommon and risk generating characteristics. Items that were “less difficult” were ordinal items for which the first ranks had difficulty values under 0. However, their later ranks had difficulties over 0, suggesting they captured a wide range of risk. Therefore, the age at release, unrelated and stranger victim, and prior arrest/sentencing occasions items could be more common in offenders with a lower sexual recidivism risk, in contrast with very difficult items such as noncontact sexual offenses and juvenile and adult sexual offenses, which would be very rare in men with lower sexual recidivism risk.

## Discussion

The objective of the current article was to apply an MIRT model to improve our understanding of tools designed to assess the reoffending risk of men adjudicated for a sexual offense, by examining the latent structure of the Static-2002R. The Static-99R and Static-2002R are considered by many practitioners as the gold standards in assessing the baseline, static risk of sexual reoffending (Archer et al., 2006; Bourgon et al., 2018; Jackson & Hess, 2007; Kelley et al., 2020; Neal & Grisso, 2014). Therefore, understanding their latent structure is not only useful for sexual offending theorization but also paramount for sexual offender risk assessment practice. Psychometric analyses beyond predictive validity comprise many potential advantages, namely, moving the field from a unidimensional conceptualization of risk toward a multidimensional model where multiple psychological constructs intertwine to lead to recidivism. In other words, it enables the study of the building blocks of recidivism with finer granularity than if a single risk dimension was used.

This echoes developments in the assessment of psychopathy, where varying scores on the two factors and four facets of the *Psychopathy Checklist–Revised* (PCL-R; Hare, 2003; Neumann et al., 2007) produce different clinical entities (e.g., high scores on Factor 1 and low scores on Factor 2 depict the “white-collar psychopath,” which is different from persons who would obtain high scores on both factors). In the case of the three-factor model of sexual violence risk, high scores on Persistence/Paraphilia and low scores on Youthful Stranger Aggression and General Criminality might produce a clinical portrait similar to that of the fixated pedophile of Groth et al. (1982), while high scores on General Criminality and low to medium scores on Persistence/Paraphilia and Youthful Stranger Aggression might echo Knight and Prentky’s (1990) opportunistic sexual aggressor of women. The mapping of different combinations of scores on the three-factor model and existing typologies of individuals that sexually offended would have to be validated by future studies, but they nevertheless open an interesting interface between risk assessment research and on-the-ground clinical work where typologies usually play a more important role.

Finally, the present study comprises advantages associated with the use of MIRT rather than traditional factor analysis only. Using MIRT models enables the reduction of circular dependency (and thus improves generalizability), gives insight on the calibration of items with the difficulty and discrimination parameters, and allows the comparison of results with traditional EFA models.

### Factor Structure

As to the factor structure, analyses revealed that MIRT results were very close to EFA results, as well as those from other empirical studies of the latent structure of the Static-99R and Static-2002R (e.g., Brouillette-Alarie et al., 2016; Brouillette-Alarie & Proulx, 2013; Roberts et al., 2002; Seto, 2005). Fit indices of our EFA converged on three factors very similar to those found in Brouillette-Alarie et al. (2016), which is not surprising considering that the same dataset was used in Brouillette-Alarie et al. (2016) and the current study. However, the results of Brouillette-Alarie et al. (2016) were based on nonredundant items from the Static-99R and Static-2002R, while the current study comprised Static-2002R items only. The three factors extracted encompassed the two main dimensions of sexual recidivism risk: (a) sexual criminality, indicative of atypical sexual interests and/or sexual preoccupations, and (b) general criminality, indicative of psychopathic traits and/or an antisocial lifestyle (Brouillette-Alarie & Hanson, 2015; Doren, 2004).

The third dimension was centered around young age, juvenile sexual offenses, and the sexual abuse of unrelated/stranger victims. The scientific consensus about this third dimension is currently unclear, some attributing this construct to demographics (younger offenders are unlikely to have access to intrafamilial victims, for example, their own children; Brouillette-Alarie et al., 2016), some to intent to harm victims (Brouillette-Alarie et al., 2018; Lehmann et al., 2014; Roberts et al., 2002), and others to sexual offender types (sexual aggressors of women are, on average, younger than sexual aggressors of children and use more coercion in their sexual offenses; Knight & Thornton, 2007). Even though the factor may not represent a psychologically meaningful construct, it may correlate with multiple psychological and physiological traits, as does aging. Indeed, the link between age and crime is a classic in criminology, dating to the early studies of Quételet (1835) and having been replicated ever since with offenders with or without a history of sexual crime (Hanson, 2002; Hirschi & Gottfredson, 1983; Moffitt, 1993; Sampson & Laub, 2003). As mentioned by Barbaree et al. (2009), age could have an interaction effect with sexual and general criminality. In their study, sexual criminality items were more predictive of sexual recidivism for older individuals, while general criminality items were more predictive of sexual recidivism for younger individuals. This would fit with MIRT results from this study, where the “age at release” item obtained very high discrimination slopes for both Persistence/Paraphilia and Youthful Stranger Aggression, but in opposite directions. This indicates that for Persistence/Paraphilia, older ages are more discriminatory, while for Youthful Stranger Aggression, younger ages are more discriminatory. Even though this unusual pattern should not be a sufficient justification to advocate for the removal of age in the Static-2002R, as the item is certainly risk relevant (Hanson, 2002), it may suggest that future factor analyses or MIRT evaluations of the scale may be better served by removing the age item a priori from the solution.

Our factor solution was discordant with that of Jung et al. (2017), the only other factor analysis study of the Static-2002R as a whole. In their study, two factors were retained because three- and four-factor solutions comprised Heywood cases (loadings greater than 1.0; Heywood, 1931). Heywood cases are often indicative of insufficient sample size or too many factors extracted, which motivated Jung et al. (2017) to settle on two dimensions. Interestingly, in their two-factor solution, the age at release and juvenile and adult sexual offenses items did not load on any factor, suggesting they may have belonged to a third, unextracted dimension. In addition, among their factor retention criteria, parallel analysis—arguably the most robust criterion (O’Connor, 2000; Schmitt, 2011)—suggested three factors.

### MIRT

MIRT analyses indicated that all Static-2002R items, except for juvenile and adult sexual offenses, performed more effectively (i.e., changed more quickly in probability) in the factor in which EFA classified them. The juvenile and adult sexual offenses item had a significantly higher discrimination slope in Youthful Stranger Aggression than in Persistence/Paraphilia, which was not entirely surprising considering that EFA results highlighted that this item cross-loaded on both Youthful Stranger Aggression and Persistence/Paraphilia. In addition, apart from the previously discussed “age at release” item, no Static-2002R item had substantial (moderate or more) negative discrimination in a factor. This suggests, along with the low but positive correlations between factors, that Static-2002R dimensions are different psychological entities that nevertheless incrementally contribute to risk of future sexual offending. It would remain to be seen if and how these dimensions interact to lead to recidivism, which would make an interesting avenue for future research, as the current study did not account or test for moderation effects.

In sum, results indicate a good convergence between EFA loadings and MIRT discrimination slopes—two different but complementary families of statistical analyses—and further contribute to cement the factor structure of the Static-2002R.

#### Multidimensional Item Discrimination and Difficulty

The MIRT model gave valuable information on the performance of Static-2002R items. Knowing item-level parameters has numerous potential advantages for actuarial scale development, namely, by suggesting avenues to improve item weighting according to discrimination and difficulty. Scaling items according to their psychometric properties rather than giving them equal weight (usually 1 point) has the potential to improve the predictive validity of scales and make them more face valid, as not all risk factors are likely to be equally related to recidivism. There are debates on the tangible benefits of differentially weighting items, with some results indicating that complex combinations rarely outperform the simple summing of dichotomous items (Ghiselli et al., 1981; Grann & Långström, 2007; Silver et al., 2000). However, differential weighting has its greatest impact when there is a wide variation in weighting values, little intercorrelation between items, and only a few items (Ghiselli et al., 1981; Kline, 2005). Considering that actuarial scales usually comprise few nonredundant items, they could benefit from differential weighting.

Concerning multidimensional discrimination (*A*), none of the Static-2002R items had values that were unsatisfactory. Only noncontact sexual offenses, male victim, and unrelated and stranger victim—sexual violence–related items—showed moderate rather than very high discrimination. General Criminality items were found to be very discriminant, suggesting that the BARR-2002R, which contains those items plus age, might display particularly good psychometric properties in IRT analyses.

All Static-2002R items except age at release, unrelated and stranger victim, and prior arrest/sentencing occasions were found to be difficult (multidimensional difficulty [*B*] > 0). The first ranks of these ordinal items had difficulty values under 0 and their later ranks over 0, suggesting they captured a wide range of risk. As mentioned by Giguère and Lussier (2016), less difficult items are not a problem by themselves, as actuarial scales should aim to have difficult and less difficult items to cover all levels of the risk continuum. Our analyses suggested that in the Static-2002R, age at release, unrelated and stranger victim, and prior arrest/sentencing occasions were the items most likely to cover the lower end of the risk continuum and, thus, to be found among lower risk individuals. Difficult items included noncontact sexual offenses, juvenile and adult sexual offenses, and, to a lesser extent, male victim, young, unrelated victims, and the later ranks of prior arrest/sentencing occasions and prior sexual offenses. Therefore, endorsement of these items is more likely to be found among higher risk individuals and should be rare among lower risk individuals. In the Static-2002R, difficulty mostly manifested itself in sexual violence items, which is consistent with the scarcity of sexual offenses in criminal records compared with nonsexual offenses. Indeed, men with a history of sexual crime are more likely to reoffend with a nonsexual offense than a sexual crime (Hanson & Bussière, 1998; Hanson & Morton-Bourgon, 2005) and are more likely to be generalists than “sexual specialists” in their criminal careers (D. A. Harris et al., 2011; Lussier et al., 2005). In future developments of the Static-2002R, it might be worthwhile to test whether improving the weighting of the “juvenile and adult sexual offenses” and “noncontact sexual offenses” items could lead to improvements in predictive validity, as these items were the most difficult and worth 1 point only in the scale.

In sum, MIRT analyses did not single out any Static-2002R item as problematic, echoing the findings of Helmus and Thornton (2015), which tested the predictive and incremental validity of Static-99R and Static-2002R items in a meta-analysis. They found that only index nonsexual violence proved problematic—an item absent from the Static-2002R and, consequently, from our study. Therefore, the current research attests to the relevance of all Static-2002R items. Even though more explicit coding rules or a reworking of the structure of less discriminative items (noncontact sexual offenses, male victim, and unrelated and stranger victim) might potentially improve the scale, there are no impetus or warning flags given that items demonstrated at worst moderate discrimination levels.

As to implications of the MIRT model for future research, apart from suggesting avenues to re-weight items, it also brings the complementary question of the convergence between MIRT parameters and risk levels. If two individuals are scored positively on four 1-point items of the Static-2002R and thus obtain a score of 4, are their risk levels the same if the items endorsed are not the same and have varying levels of difficulty? Current CTT models (and the Static-2002R) would assume that their risk is similar, unlike θ positioning on an IRT model. Future studies can examine whether these two hypothetical individuals actually differ on recidivism risk. That said, we must be cognizant that the meaning of item discrimination and difficulty might not be the same in risk scales than in mathematical tests from the field of education. Success on a complicated mathematical problem usually implies success on an easier problem. In the context of risk scales, this assumption might not hold true, especially in multidimensional scales like the Static-2002R (hence the use of MIRT). For example, endorsing the “noncontact sexual offenses” is likely to come with endorsement of the less difficult “prior sexual offenses” item, as committing a noncontact sexual offense automatically means that you have committed at least one prior sexual offense (unless the noncontact sexual offense was committed in the index offense). However, it does not mean endorsement of the “male victim” or “prior nonsexual violence” items—especially the latter one, as it is part of another dimension. This implies that even though MIRT opens numerous interesting avenues for risk scale development, these avenues need to be concretely tested for usefulness before discarding previous advances in the field anchored in CTT models.

### Limitations

The main limitation of the current study relates to the item preparation that was necessary before submitting the scale for EFA and MIRT. Collinearity concerns led us to merge items (e.g., unrelated/stranger victim) and discard others (high rate of sexual offending), which implies that the Static-2002R was not entered “as is” in latent variable analyses. This may have influenced the outcoming factor structure and deprives us of data on the high rate of sexual offending item. For example, even though it would be anticipated to find this item in Persistence/Paraphilia, it may have significantly loaded on General Criminality, a factor also characterized by criminal repetition. A necessary step before integrating dimensional scores in the Static-2002R would be to perform an EFA and/or CFA with items entered without modifications and compare results with those obtained in the current study. Encouragingly, recent studies on sadism have shown consistency of findings across samples and analytical strategies whether item preparation was performed or not (Longpré et al., 2020; Stefanska et al., 2019; Yoon et al., 2019).

### Implications for Practice

The implications for practice of the current study are threefold. First, as mentioned above, results confirm the relevance of using the Static-2002R to assess the risk of adult men with a history of sexual crime. Substantial literature already exists on the reliability and predictive validity of the scale (Babchishin et al., 2012; Hanson et al., 2010; Phenix & Epperson, 2016; Tully et al., 2013), and this study further attested to its psychometric properties with items performing well under MIRT scrutiny. Other actuarial scales in the field, such as the Level of Service/Case Management Inventory (LS/CMI; Andrews et al., 2004), were not so lucky (Giguère & Lussier, 2016).

Second, this study emphasized the importance of integrating dimensional scores in the Static-2002R. Our results indicated that recidivism risk in men who sexually offend was multidimensional: The EFA identified a three-factor solution; these factors did not share a substantial amount of variance; and there was no higher order risk dimension that encompassed the three factors. In other studies, these dimensions showed predictive and convergent validity patterns that were quite differentiated (Brouillette-Alarie et al., 2016, 2018). Considering that the nomological networks of these constructs seem dissimilar, they may not represent the same psychological (or physiological) constructs, which emphasizes the need to switch to a dimensional scoring of risk in actuarial instruments. Such a change could enable improvements in predictive validity depending on the outcome of interest. For example, the BARR-2002R outclasses the predictive validity of the Static-2002R for nonsexual recidivism because it discards a dimension (sexual criminality) irrelevant to that outcome (Babchishin et al., 2016). Dimensional scoring would also open the door for future examinations of the nomological networks of these constructs, which could retroactively lead to further improvements in predictive validity.

Third, studies that evaluated the Static-2002R’s cross-cultural validity also highlighted the importance of dimensional scoring. Indeed, recent studies have shown that the Static-99R was similarly predictive of recidivism for White, Black, Hispanic, and Indigenous individuals with a history of sexual crime (Lee & Hanson, 2017; Lee, Hanson, & Blais, 2020). However, for the Static-2002R, when comparing Caucasian and Indigenous offenders, the Static-2002R failed to be predictive for Indigenous participants because the Persistence/Paraphilia and Youthful Stranger Aggression constructs were not predictive (Lee, Hanson, & Blais, 2020). Thus, static risk dimensions comprising sexuality-related items were not predictive compared with General Criminality items. Another relevant result was found in a study that compared motivations for sexual offending among White and Black participants (Lee, Hanson, Calkins, & Jeglic, 2020). Results indicated that White men had higher paraphilic and lower antisociality scores compared with Black men. In both cases, had the authors failed to explore risk constructs in addition to total scores, important nuances would have been lost, including opportunities to improve treatment tailoring in the future.

In sum, considering that in the current study the factor structure of the Static-2002R was consistent in EFA and MIRT and that similar structures were found in other studies of the Static-99R/2002R (Brouillette-Alarie et al., 2016; Brouillette-Alarie & Proulx, 2013; Roberts et al., 2002; Seto, 2005), there is now sufficient evidence for integrating dimensional scores in the Static-2002R. For the prediction of sexual recidivism, all three constructs—thus all Static-2002R items—would likely be used. For nonsexual recidivism, it would remain to be seen if the prediction of nonsexual recidivism would be better served by using the BARR-2002R (General Criminality plus the age item) or the sum of General Criminality and Youthful Stranger Aggression. In any case, the existing five subscales of the Static-2002R should be replaced by those found in studies of the latent structure of the scale, as empirical results do deviate from the initial division proposed by creators of the instrument.

## Appendix A

**Table A1.** Static-2002R Items Used in This Study.

Item no.	Original item no.	Risk factor	Codes	Score
1	1	Age at release	18–34.9	2
			35–39.9	1
			40–59.9	0
			60 or older	−1
2	2	Prior sexual offenses	None	0
			1	1
			2, 3	2
			4 or more	3
3	3	Juvenile (<18) and adult sexual offenses	No	0
			Yes	1
4	5	Noncontact sexual offenses	No	0
			Yes	1
5	6	Male victim	No	0
			Yes	1
6	7	2+ young (<12) and 1+ unrelated victims	No	0
			Yes	1
7	8 and 9	Unrelated and stranger victim	No	0
			Yes (unrelated)	1
			Yes (stranger)	2
8	10 and 11	Prior arrest/sentencing occasions	0	0
			1–2	1
			3–13	2
			14 or more	3
9	12	Community supervision violation	No	0
			Yes	1
10	13	Years free prior to index offense	No	1
			Yes	0
11	14	Prior nonsexual violence	No	0
			Yes	1

*Note.* The “high rate of sexual offending” item (original item no. 4) was dropped in this study because of its high correlation with the “prior sentencing occasions for sexual offenses” item (*r* = .95).

## Appendix B

**Table B1.** Polychoric/Tetrachoric Correlations Between Static-2002R Items (N = 2,569).

Items	1	2	3	4	5	6	7	8	9	10	11
1	1.00	—	—	—	—	—	—	—	—	—	—
2	−.11	1.00	—	—	—	—	—	—	—	—	—
3	.33	.62	1.00	—	—	—	—	—	—	—	—
4	−.08	.46	.09	1.00	—	—	—	—	—	—	—
5	−.27	.33	.19	.23	1.00	—	—	—	—	—	—
6	−.15	.47	.34	.32	.56	1.00	—	—	—	—	—
7	.25	.37	.39	.28	.04	.34	1.00	—	—	—	—
8	<.001	.56	.42	.14	−.01	.04	.22	1.00	—	—	—
9	.24	.48	.32	.09	−.07	<.001	.24	.80	1.00	—	—
10	.33	.53	.45	.13	−.01	.13	.41	.71	.76	1.00	—
11	.11	.21	.17	−.08	−.17	−.20	.16	.67	.65	.57	1.00

*Note.* 1 = age at release; 2 = prior sexual offenses; 3 = juvenile and adult sexual offenses; 4 = noncontact sexual offenses; 5 = male victim; 6 = young, unrelated victims; 7 = unrelated and stranger victim; 8 = prior arrest/sentencing occasions; 9 = community supervision violation; 10 = years free prior to index offense; 11 = prior nonsexual violence.
